# Silver Compositing Boosts Water Electrolysis Activity and Durability of RuO_2_ in a Proton‐Exchange‐Membrane Water Electrolyzer

**DOI:** 10.1002/smsc.202300055

**Published:** 2023-07-18

**Authors:** Jiayi Tang, Yijun Zhong, Chao Su, Zongping Shao

**Affiliations:** ^1^ WA School of Mines: Minerals, Energy and Chemical Engineering (WASM-MECE) Curtin University Perth WA 6102 Australia; ^2^ School of Energy and Power Jiangsu University of Science and Technology Zhenjiang 212100 China

**Keywords:** catalytic sites utilization, electrode engineering, oxygen evolution reaction, proton-exchange-membrane water electrolyzers, water electrolysis

## Abstract

Proton‐exchange‐membrane water electrolyzers (PEMWEs) are of particular interest for green hydrogen production, where the oxygen evolution reaction (OER) at the anode largely determines the overall efficiency. Up to now, only ultrafine IrO_2_ catalyst gives desirable performance, while its scarcity and high cost inhibit the widespread application. RuO_2_ catalyst is the most promising alternative, while its practical application is greatly hindered by poor durability. Herein, the greatly boosted performance of conventional sub‐micrometer RuO_2_ by compositing with Ag is reported, and both the morphology of Ag and the compositing way significantly affect the electrolysis performance. The PEMWE fabricated with a two‐layer RuO_2_/Ag nanowire (NWs) composite anode achieves 1.77 A cm^−2^ at 2.00 V, due to a prominent 44.6 times increase of the electronic conductivity, which greatly improves the catalyst utilization. In addition, mass transportation at high‐current‐density region is enhanced due to the highly porous feature of Ag NW layer. Long‐term stability under high current density of 1 A cm^−2^ for 100 h is proved with the composite anode, due to the suppressed degradation of RuO_2_ by silver compositing. This work may accelerate the widespread commercialization of PEMWEs by providing a new way for developing IrO_2_‐free anode.

## Graphical abstract

A novel RuO_2_/Ag composite electrode with enhanced water electrolysis performance in practical water electrolyzers is developed. Reduced reaction barriers and enhanced electrode activity and durability are achieved by silver compositing, due to the improved electrode reaction kinetic and catalytic sites utilization with the improved electron conductivity and enhanced mass transportation with the increased porosity of the electrode.

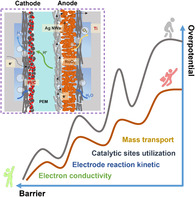

## 1 Introduction

Low‐cost large‐scale generation of green hydrogen is an important step toward the hydrogen economy, while water splitting from renewable energy is believed to be the most sustainable way considering the wide availability of seawater and renewable solar and wind energy.^[^ ^1^, ^2^ ^]^ For above reasons, during the past several years, there are tremendous interests in developing highly efficient and durable electrodes for proton‐exchange‐membrane water electrolyzers (PEMWEs) to achieve sustainable green hydrogen production, owing to the considerably high chemical and mechanical stability and high conductivity of proton exchange membranes (PEMs), relatively mature electrode preparation technique, zero‐gap cell configuration, as well as the high operating current density. During the operation of an PEMWE for overall water electrolysis, hydrogen evolution reaction (HER) and oxygen evolution reaction (OER) take place at the cathode and anode, respectively, as follows

(1) $$\text{Anode OER}:\;2H_{2}O\to 4H^{+}+O_{2}+4e^{-}$$

(2) $$\text{Cathode HER}:\;4H^{+}+4e^{-}\to 2H_{2}$$

It has been well recognized that the anodic OER process under acidic environment is the key factor that limits the overall water electrolysis efficiency in PEMWEs.^[^ ^3^ ^]^ Up to now, the main challenge for the widespread application of PEMWE technology is still the high cost and insufficient activity and durability associated with the OER electrode. Due to the acidic environment and highly oxidizing operating conditions in PEMWEs, most of the electrocatalysts containing non‐noble metal elements only exhibit poor catalytic activity and durability.^[^ ^4^, ^5^ ^]^ The state‐of‐the‐art OER electrocatalyst at current stage is still the high‐surface‐area IrO_2_.^[^ ^6^ ^]^ However, the scarcity and high cost of Ir (4600 USD/Oz) significantly impede the large‐scale application of PEMWEs. Alternative OER electrocatalysts applicable to the PEMWE operating conditions with high activity, favorable stability, and low cost are desperately needed.

Among various electrocatalysts designed for OER under acidic environment, RuO_2_ has received particular attention, which consistently represents the best‐performing catalyst showing even higher intrinsic activity than IrO_2_ for OER, but the price of Ru (465 UD/Oz) is only of around 10% of Ir.^[^ ^7^, ^8^ ^]^ However, the more severe dissolution and degradation of RuO_2_ during the acidic OER is an obvious drawback which limits its wide application.^[^ ^9^ ^]^ Although reducing the particle size and increasing the surface area of RuO_2_ may increase the accessible active sites of the catalyst and achieve better electrode performance; however, it generally suffers from higher decay rate due to the accelerated corrosion of electrocatalyst from the increased water–electrode contact area.^[^ ^10^ ^]^ On the other hand, in terms of the device‐level water electrolysis, to improve the energy efficiency and reduce the system cost, maximizing the catalytic active sites utilization over the entire electrode is highly desired.^[^ ^11^, ^12^ ^]^ Unfortunately, the device‐level high performance of PEMWEs reported in literature is usually based on electrodes with a high Ir or Ru loading of 1.5–3 mg cm^−2^.^[^ ^13^, ^14^, ^15^ ^]^ Such a high catalyst loading was applied mainly to compensate the low‐catalytic‐sites utilization within the electrodes,^[^ ^16^ ^]^ which has been attributed to the insufficient electronic conductivity of the catalyst layer in water^[^ ^14^ ^]^ or the ineffective interfacial contact between the porous transport layer and the catalyst layer.^[^ ^17^ ^]^

To increase catalyst utilization within the electrode, theoretical analysis and experimental efforts have been mostly focused on optimizing the porous transport layer to improve the interfacial contact with the catalyst layer.^[^ ^18^, ^19^ ^]^ However, the improvement as brought by the contact interface could be limited, while few works are involved in essentially improving the catalytic site utilization within the catalyst layer itself. The catalyst layer, as providing actual sites for the electrochemical reaction, is bound to directly influence the electrode reaction performance. Besides, catalyst layers as formed by the catalyst‐coated membrane (CCM) technique with typical microporous structures are often accompanied by the greater mass transportation resistance during water electrolysis, especially for operating under high current density in PEMWEs, which could be due to the difficult water reactant infiltration and gas products’ release from the electrode.^[^ ^20^ ^]^

Ag, generally mined as a byproduct of gold,^[^ ^21^ ^]^ exhibits the highest conductivity among all the metals and has been extensively used as current collector in solar cells,^[^ ^22^ ^]^ while the price of which is only about 1% of gold. Here, we demonstrate that for the first time the catalytic activity and durability of coarse RuO_2_ catalyst in sub‐micrometer size can be greatly improved through compositing with Ag, and both the morphology and the compositing way of Ag for forming the catalyst layer would greatly influence the utilization of RuO_2_ catalyst, therefore affecting its practical catalytic performance in PEMWEs. In particular, a two‐layer‐structured RuO_2_/Ag composite anode as formed with a porous Ag layer composed of Ag NWs, when fabricated as the anode in a PEMWE, was able to deliver an electrolysis current of 1.77 A cm^−2^ at 2.00 V, which achieving an increase of 34% as compared with the pristine RuO_2_ anode. The special interweaving structure of Ag NWs in the electrode and the two‐layer electrode configuration effectively improved the catalytic site utilization of RuO_2_ catalyst due to a prominent 44.59 times increase of the electronic conductivity of the anode and facilitated the mass transportation for water electrolysis at high current density. In addition, we found that the Ag compositing alleviated the performance degradation caused by the loss of RuO_2_ during water electrolysis and allowed the PEMWE to maintain long‐term stability at a high catalytic site utilization by operating under 1 A cm^−2^ high current density for 100 h without obvious performance decay. The role of Ag NWs in the catalyst layer for boosting the water electrolysis performance and durability in PEMWEs was elucidated in this work. This work may provide a new way for the development of efficient IrO_2_‐free electrodes for PEMWEs, which may accelerate the large‐scale commercialization of this technology for green hydrogen generation.

## 2 Results and Discussion

Among various research works regarding the development of OER electrocatalysts, high‐specific‐area materials with fine structures have been highly valued to expose more effective sites and achieve desirable electrocatalysis performance.^[^ ^23^ ^]^ While for the RuO_2_ catalyst, its stability remains a big concern despite its high activity, and the practical application of the high‐surface‐area RuO_2_ catalyst poses a more significant challenge to its dissolution and accelerated performance decay due to the higher catalyst–reactant contact interface. In order to break the stability–activity seesaw of RuO_2_ catalyst and make it applicable for highly efficient and stable water electrolysis, the proper use of the relatively‐stable coarse RuO_2_ catalyst with lower surface area but to improve the catalyst utilization can be a solution.

For electrodes composed of oxide electrocatalysts with modest electronic conductivity, the insufficient catalyst utilization is often manifested due to the ineffective electron conductance.^[^ ^24^, ^25^ ^]^ Ag, as the most conductive metal, was selected to composite with RuO_2_ to improve the electronic conductivity of the catalyst layer. As a proof of concept, commercially available sub‐micrometer RuO_2_ catalyst was selected, so that the conclusions do not rely on any specially designed catalyst structure and can be generally applicable. The mass specific surface area of the RuO_2_ catalyst as determined by the Brunauer–Emmett–Teller (BET) measurement was 10.8 m^2^ g^−1^, much lower than the commonly used high‐specific‐area RuO_2_ or IrO_2_ in 50–100 m^2^ g^−1^.^[^ ^26^, ^27^, ^28^ ^]^ The morphology of the RuO_2_ catalyst was characterized by transmission electron microscopy (TEM) (Figure S1a, Supporting Information), which showed irregular dimensions in the range of 0.2–0.6 μm, and the Ag NWs as characterized were in a uniform dimension of 60 nm in diameter (Figure S1b, Supporting Information). The RuO_2_ particles were suggested to be dense in nature as supported by the nitrogen adsorption isotherm, as shown in Figure S2, Supporting Information, and the active sites were mainly from the outer surface of the coarse particle. Different compositing ways for forming the anode catalyst layer using Ag NWs with sub‐micrometer RuO_2_ particles have been explored. Besides, to further demonstrate the structural advantage of Ag NWs, spherical Ag nanoparticles (NPs) were selected as an alternative to Ag NWs and also investigated for forming the anode catalyst layer.

**Figure 1:**
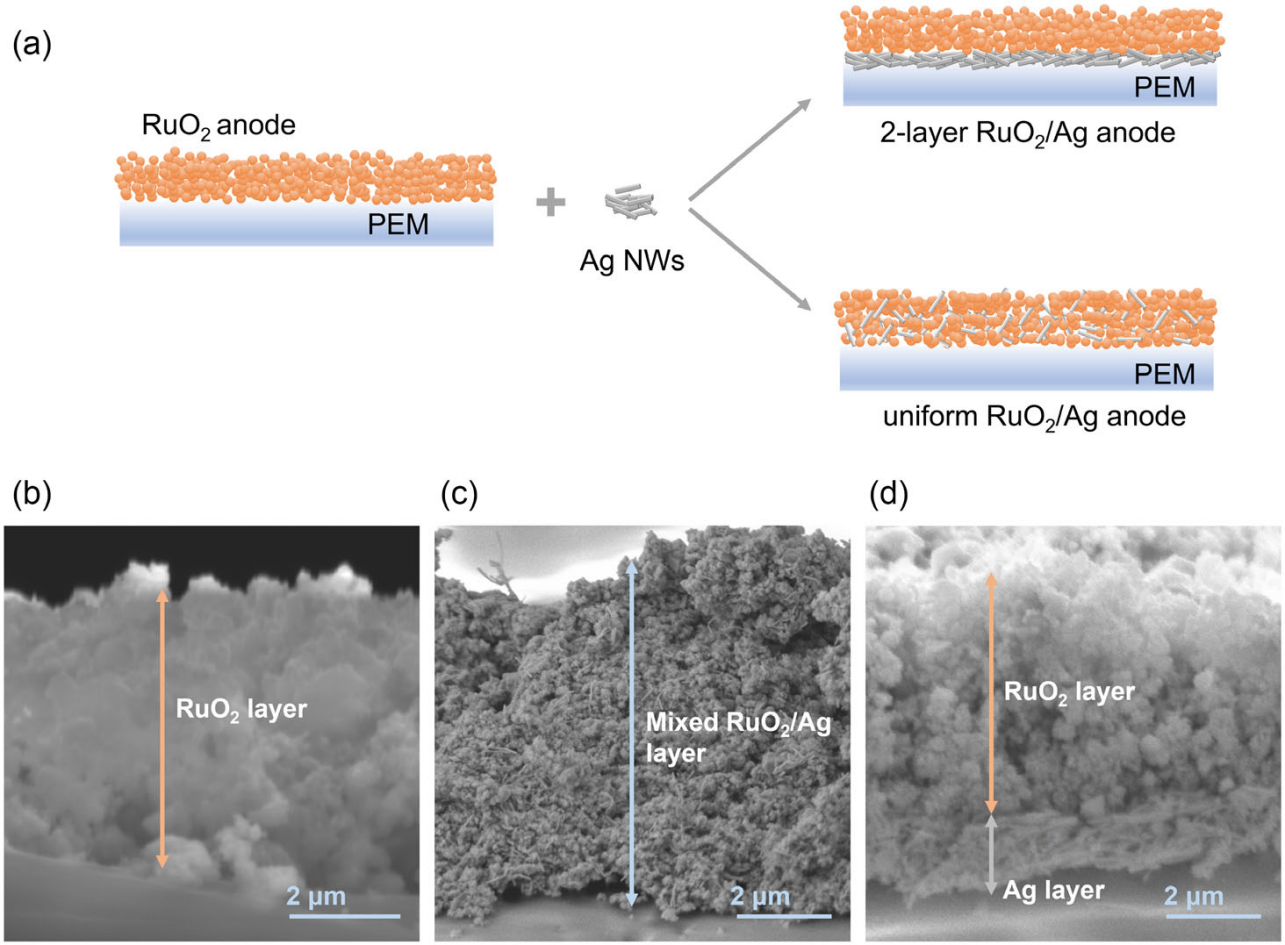
Catalyst layers preparation and characterization. a) Schematic illustration of the anode configurations with RuO_2_ catalyst and the Ag NWs on the PEM. b–d) Cross‐section SEM images of the RuO_2_ anode (b), uniform RuO_2_/Ag anode (c), and two‐layer RuO_2_/Ag anode (d)_2_/Ag anode.

First, Ag NWs are prone to form a crosslinked network and thus serves as an effective electronic conductor to facilitate a uniform distribution of current within the catalyst layer. On the other hand, the interwoven stacking of Ag NWs is likely to form a porous‐rich catalyst layer structure, which is conducive to the mass transportation behaviors during the electrode reaction especially at high current density. In order to give full play to the effect of Ag NWs in the catalyst layer, we explored the fabrication of RuO_2_/Ag composite anodes by introducing Ag NWs in two different ways, as illustrated in **Figure** **1**a. Spraying coating was applied as a facial way to control the process of catalyst deposition and obtain a relatively uniform catalyst layer. In one approach, Ag NWs were uniformly dispersed throughout the catalyst layer by premixing with RuO_2_ catalyst before spray deposition, the catalyst layer as formed is labeled as uniform RuO_2_/Ag anode. The cross‐section scanning electron microscopy (SEM) image of the uniform‐RuO_2_/Ag anode (Figure 1c) shows that the average thickness of the catalyst layer was measured to be ≈7.12 μm. Compared with the pristine RuO_2_ anode of ≈5.04 μm (Figure 1b), an obvious increase in the thickness could mean a higher porosity of the catalyst layer. While in the other way, Ag NWs were first deposited onto the membrane to form a thin base layer, then RuO_2_ catalyst was sprayed to form a bilayer anode structure, which is labeled as two‐layer RuO_2_/Ag anode. As shown in Figure 1d, the Ag base layer as composed of Ag NWs is of an average thickness of 1.72 μm, taking into account postdeposited RuO_2_ layer, the total thickness of the two‐layer RuO_2_/Ag anode is ≈6.84 μm. This configuration, compared with the uniform RuO_2_/Ag anode, is composed of two respectively continuous RuO_2_ layer and Ag layer, and the two layers are tightly combined to form a seamless interface by hot pressing.

**Figure 2:**
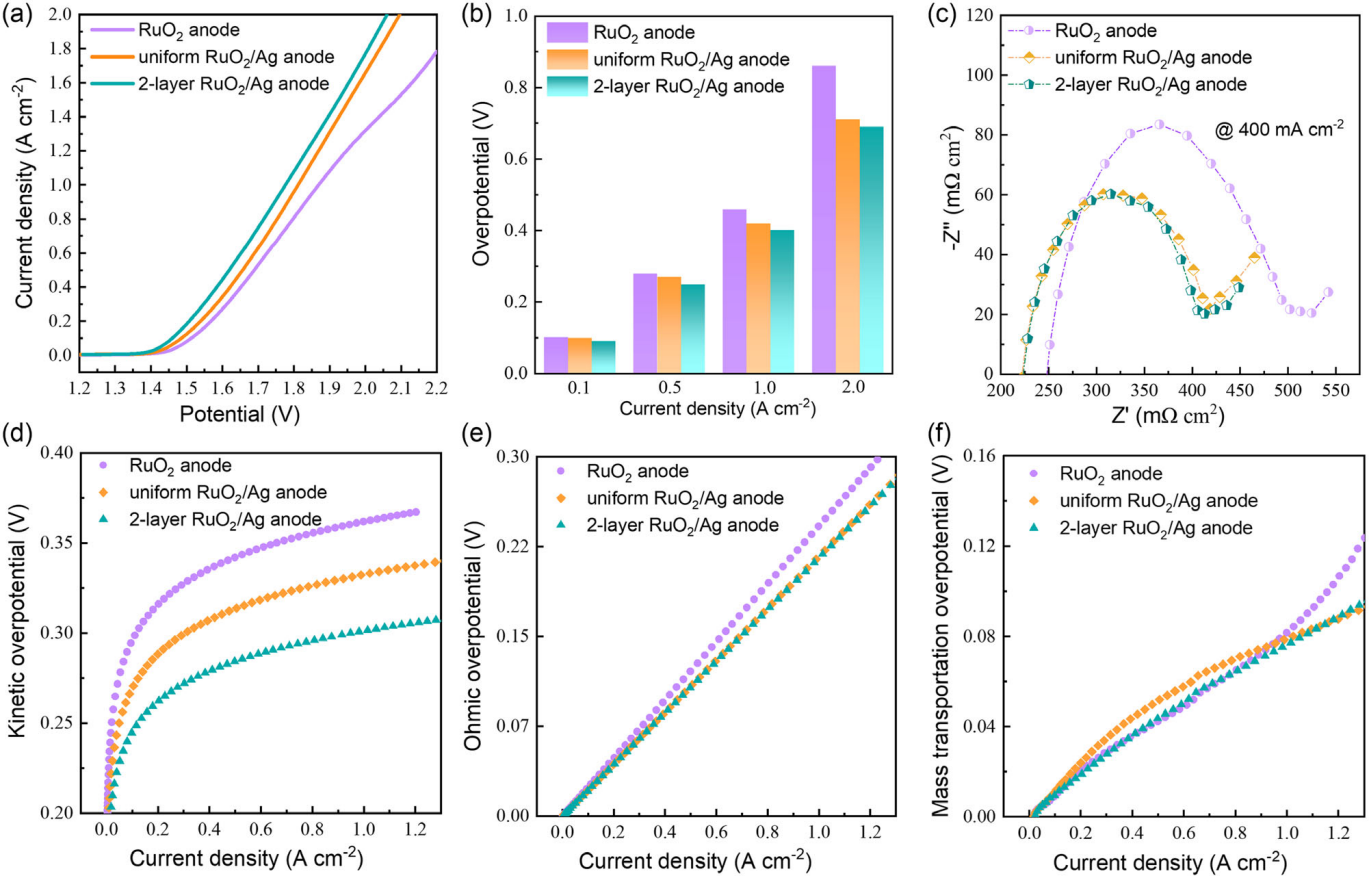
Polarization performance of the PEMWEs fabricated with RuO_2_ anode, uniform RuO_2_/Ag anode, and two‐layer RuO_2_/Ag anode. a) Polarization curves for the overall water electrolysis. b) Overpotentials for reaching different electrolysis current densities. c) Nyquist plots from galvanostatic EIS measurements at 400 mA cm^−2^. d–f) Partial polarization losses against current density of the operating PEMWEs: d) kinetic overpotential, e) ohmic overpotential, and f) mass transportation overpotential.

The influence of Ag NWs within the composite anodes based on RuO_2_ was first investigated by evaluating the water electrolysis performance in PEMWEs. The polarization curves representing the overall water electrolysis performance are shown in **Figure** **2**a, Figure S3 and S4, Supporting Information. The PEMWE fabricated with the two‐layer RuO_2_/Ag anode exhibited a significant performance improvement compared with the RuO_2_ anode by achieving a 1.77 A cm^−2^ electrolysis current at 2.00 V. The as‐fabricated PEMWE presents an obvious advantage compared with the reported PEMWE performance with Ir‐ and Ru‐based anodes in literature, considering that based on the same N117 PEM assembly, a comparable and even higher electrolysis current density was achieved, indicating that the electrode as prepared may feature high catalytic site utilization. The overpotentials for reaching different electrolysis current densities are compared in Figure 2b. At small‐current‐density region as controlled mainly by the electrode reaction kinetics, the difference among the overpotentials for RuO_2_/Ag composite anodes and RuO_2_ anode is relatively small, while at large‐current‐density region, which is mainly controlled by the catalytic sites utilization and mass transportation, substantially lower overpotentials were observed with two‐layer RuO_2_/Ag anode and uniform RuO_2_/Ag anode (690 mV and 711 mV, respectively) compared with the 861 mV of RuO_2_ anode for reaching 2.00 A cm^−2^. Nyquist plots from galvanostatic electrochemical impedance spectroscopy (EIS) measurements (Figure 2c, and S5a,b, Supporting Information) performed at different current densities are presented to reflect the polarization impedances of water electrolysis in PEMWEs based on different anodes. An equivalent circuit for water electrolyzer as nominated in earlier works was introduced as a basis for the analysis of Nyquist spectra.^[^ ^29^ ^]^ The ohmic resistances as extracted from the intercept of the first semicircle with the real *Z'*‐axis at high‐frequency region are 221.9, 223.3, and 247.8 mΩ cm^2^ with the two‐layer RuO_2_/Ag anode, uniform RuO_2_/Ag anode, and RuO_2_ anode at 400 mA cm^−2^, respectively. Based on the same membrane and electrolyzer assembly conditions, the reduced ohmic resistance by Ag NWs compositing is likely to come from the optimized contact between the catalyst layer and the porous transport layer due to the slightly increased anode thickness. The main semicircle in the Nyquist spectra, which generally represents the electrode reaction impedance as dominated by the rate determining‐OER process,^[^ ^30^, ^31^ ^]^ is much smaller with the RuO_2_/Ag composite anodes, indicating that the OER reaction impedance was greatly reduced by Ag NWs compositing, as a result of the improved electron conductance within the catalyst layer.

To gain further insights into each part of polarization loss during the water electrolysis in the operating PEMWEs with different anodes, and better understand the role of Ag NWs, Figure 2d–f presents the partial polarization losses due to the reaction kinetics, ohmic resistance, and mass transportation, as decomposed from the overall polarization process. The calculations were based on Equation (1)–(5), as listed in the Supporting Information. The composition of Ag NWs was found to greatly boost the electrode reaction kinetic and reduce the kinetic overpotential. as shown in Figure 2d, especially for the two‐layer RuO_2_/Ag composite anode. To reach a representative polarization current of 1 A cm^−2^, the required kinetic overpotentials with the two‐layer RuO_2_/Ag anode and the uniform RuO_2_/Ag anode were 301.7 and 332.3 mV, respectively, which were 60.2 and 29.6 mV lower than that with RuO_2_ anode. More evidence of the enhanced electrode reaction kinetic can be found by comparing the Tafel plots (Figure S6, Table S2, Supporting Information) as constructed from the kinetic‐controlled reaction region. The two‐layer RuO_2_/Ag anode and uniform RuO_2_/Ag anode fabricated cells exhibited much lower Tafel slopes of 56.1 and 63.1 mV dec^−1^, compared with the 65.8 mV dec^−1^ for that with RuO_2_ anode. In addition, the higher exchange current densities by applying RuO_2_/Ag composite anodes signified the lower energy barriers to be overcome for charge moving across the electrode interface.^[^ ^32^, ^33^ ^]^ The composition of Ag NWs also brought about 30 mV reduction in ohmic overpotential at a polarization current of 1 A cm^−2^ (Figure 2e). The polarization loss due to the mass transportation (Figure 2f) though is not significant among the three parts, an obvious advantage could be observed with the two‐layer RuO_2_/Ag anode running over high current densities compared to the RuO_2_ anode, which is possibly due to the improved water reactant diffusion with the existence of the highly porous Ag base layer. It is noteworthy that for the uniform RuO_2_/Ag composite anode, the slightly higher mass transportation overpotential at low‐polarization current region is likely to be caused by the higher through‐plane tortuosity of the anode by mixing Ag NWs with RuO_2_ particles, and similar phenomena were also demonstrated in previous works.^[^ ^34^ ^]^ In addition, in the design of the two‐layer composite anode structure, Ag layer was used as the bottom layer (between the membrane and the RuO_2_ catalyst layer), considering not to interfere with the porous transport layer for electron transfer and mass transportation, while improving the electron conductance and mass transportation within the catalyst layer itself. This notion was also confirmed as when Ag NWs were deposited onto the RuO_2_ catalyst layer to serve as the top layer (Figure S7, Supporting Information), the advantages were no longer manifested.

**Figure 3:**
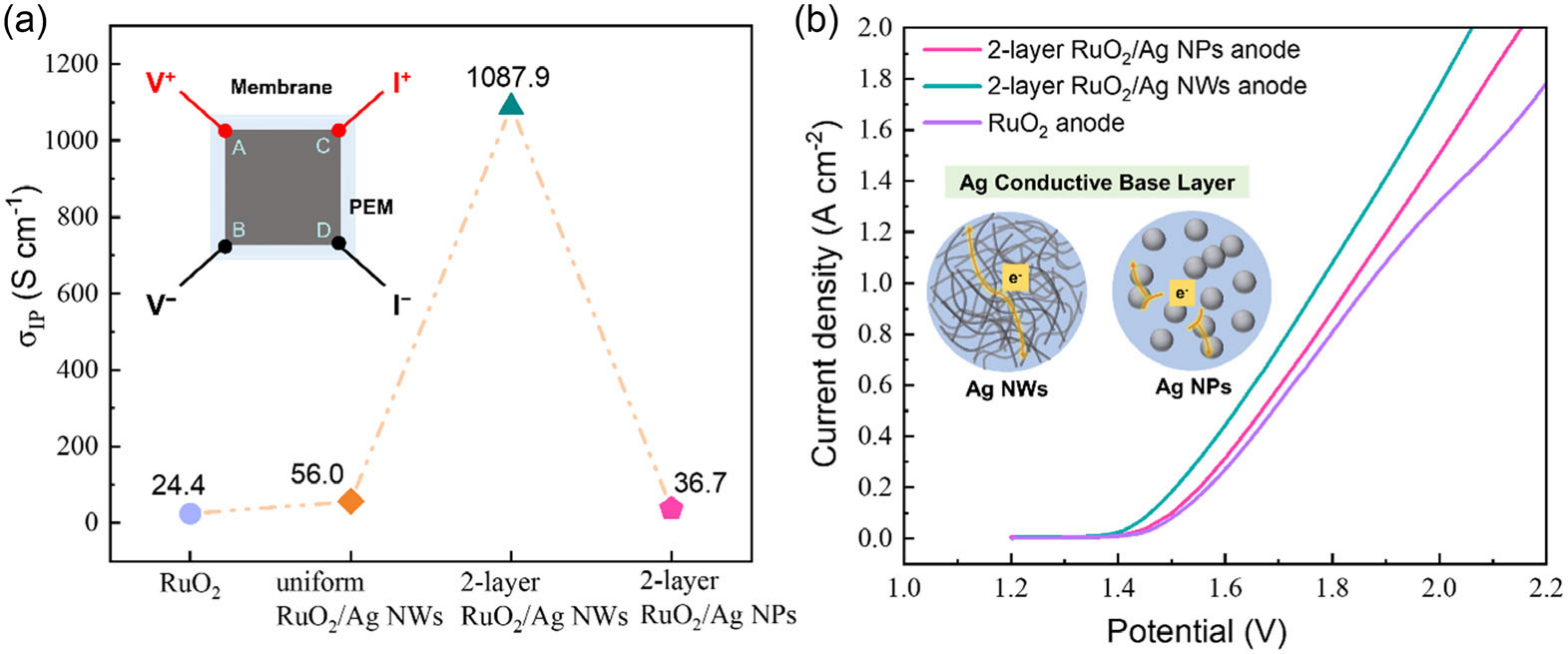
a) In‐plane electronic conductivity of different anodes as measured by the VDP method at 25 °C. The inset illustrates a four‐point probe setup for the VDP method, with the four probes placed at the periphery of the anode layer in dimension of 2 × 2 cm^2^, enabling the conductivity to be measured for the entire sheet. b) Polarization curves of the PEMWEs with different anodes for water electrolysis. The inset illustrates effective and ineffective electron conductance based on different Ag base layers composed of Ag NWs and Ag NPs, respectively.

In order to further confirm that the promotion of the electrode reaction is due to the improvement of the electron conductance of the catalyst layer by proper Ag NW compositing, and figure out the influence of Ag morphology and compositing ways, we examined the in‐plane electronic conductivity ($\sigma_{\text{IP}}$) of the several RuO_2_/Ag composite anodes as fabricated with the Ag NWs and the alternative Ag NPs, as shown in **Figure** **3**a. Van der Pauw (VDP) method (as described in details in Supporting Information) was applied.^[^ ^35^ ^]^ The in‐plane conductivity of the catalyst layer mainly reflects the lateral movement of current within the electrode, so it can be used as an important indicator to determine the catalyst utilization.^[^ ^36^, ^37^ ^]^ Besides, when the specimens are uniform layers and considered as macroscopically isotropic in nature, the in‐plane conductivity is also expected to be representative of the through‐plane values, which are considered to be difficult to measure and are error prone with ultrathin layers.^[^ ^37^, ^38^ ^]^

In stark contrast, the as‐measured in‐plane electronic conductivity of the two‐layer RuO_2_/Ag NWs anode showed a prominent increase of 44.6 times compared to that of the RuO_2_ anode. This reflects that the Ag NWs within the catalyst layer would greatly improve the catalyst utilization by enhancing the electron conductance during electrode reaction. Nevertheless, a reasonable distribution of Ag NWs is required to ensure a continuous electron conduction pathway. Thus, we can find that although the uniform RuO_2_/Ag NWs anode was formed with the same amount of Ag NWs, but only exhibited a 2.3 times higher electronic conductivity than that of the RuO_2_ anode. This moderate improvement still kept it at the same lower level, as there may still exist many isolated RuO_2_ catalyst islands, which impeded the efficient electron conductance over the entire catalyst layer. However, the unique crosslinked structure of Ag NWs is particularly beneficial for forming a continuous electron conduction pathway, as shown in Figure 3b; however, for the composite anode as‐formed with the alternative Ag spherical particles (labelled as 2‐layer RuO_2_/Ag NPs), the performance improvement as brought by this kind of electronic effect cannot be fully manifested, because of the more limited contact interface of the stacking spherical particles.

**Figure 4:**
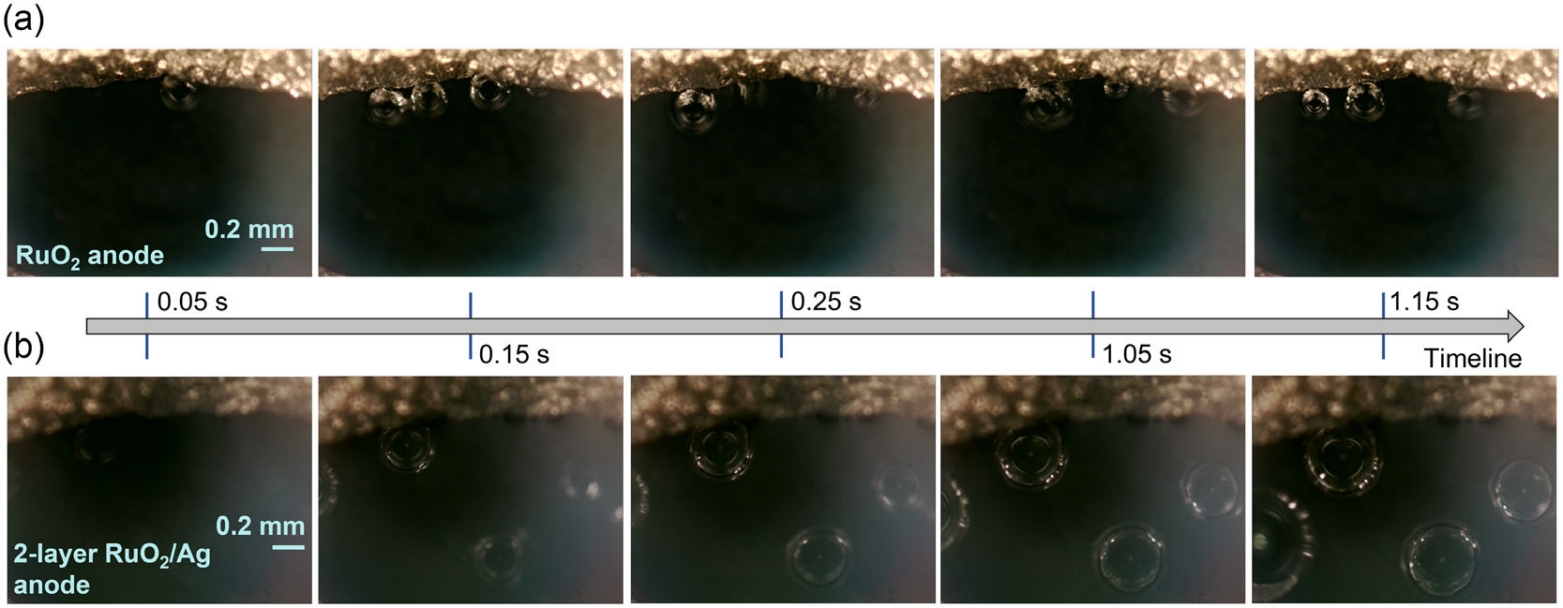
a,b) In situ observation of O_2_ bubbles generation and release during the OER from the RuO_2_ anode (a), and two‐layer RuO_2_/Ag anode (b) surfaces, respectively. The metal boundary (golden area) in the pictures is the porous Ti mesh, which is pressed on the catalyst layer (dark area) for current supply.

The catalytic site utilization would also influence the occurrence of the electrode reaction over the entire catalyst layer surface. In terms of the anode for water electrolysis, it can be reflected by the gas bubble generation and release from the electrode surface. Herein, to prove this, we applied a high‐speed camera equipped with a 5× magnification lens to capture the in situ process of O_2_ bubbles generation and release from the electrode surface during the OER. A typical open electrode setup for direct visualization is illustrated in Figure S8, Supporting Information. On the bare RuO_2_ anode, the O_2_ bubbles generation was very limited at the contact interface of the current conductor and the catalyst layer (**Figure** **4**a). This behavior was also reported in previous works, occurring under the premises of insufficient electronic conductivity of the catalyst layer or inappropriate porous structure of the diffusion layer.^[^ ^14^, ^39^ ^]^ Besides, the vigorous two‐phase mass transport at the contact interface area may cause the catalyst detachment and loss during the reaction especially at high current density and shorten the lifetime of PEMWE. While on the two‐layer RuO_2_/Ag anode, under the same applied current, the O_2_ bubbles generation occurred over the entire catalyst layer, including the blank area that was not in direct contact with the current conductor (Figure 4b), which confirmed the improved catalytic site utilization within the anode by Ag NWs’ compositing.

**Figure 5:**
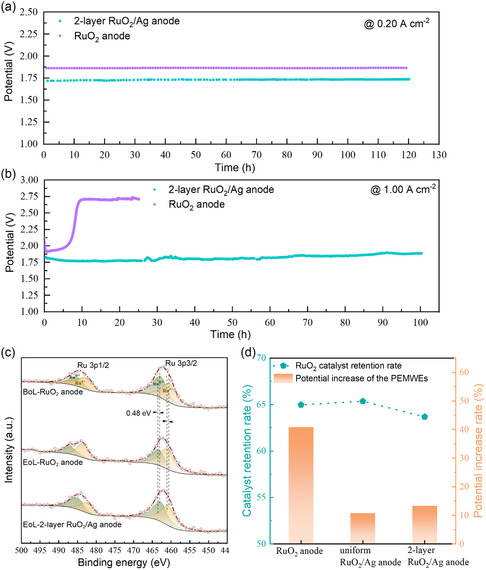
a,b) Durability tests of the PEMWEs fabricated with the two‐layer RuO_2_/Ag anode, and the RuO_2_ anode by operating under lower current density of 0.20 A cm^−2^ for 120 h (a) and higher current density of 1.00 A cm^−2^ for 100 h (b) c) Ru 3p XPS spectra of the RuO_2_ anode at the BoL (begin of life) and EoL (end of life) of the 120 h durability test at 0.20 A cm^−2^, and the two‐layer RuO_2_/Ag anode at the EoL state. d) RuO_2_ retention rate within the anode layer as measured by ICP and the potential increase rate of the PEMWE after the 100 h durability test at 1.00 A cm^−2^.

Finally, the durability of the as‐fabricated two‐layer RuO_2_/Ag anode and the RuO_2_ anode was assessed under PEMWE operating conditions as an important concern for practical application. The operations conducted both at low‐catalytic‐site utilization (small current density of 0.20 A cm^−2^) and high‐catalytic‐site utilization (large current density of 1.00 A cm^−2^) have been considered. RuO_2_ catalyst has been recognized to be unstable under acidic OER conditions, which is generally attributed to the overoxidation of RuO_2_ to the dissolvable higher‐valence RuO_4_ together with the oxidative release of lattice oxygen.^[^ ^40^, ^41^, ^42^ ^]^ By avoiding the use of nanosized RuO_2_ catalysts, which generally suffered severe performance decay within 20 h operation,^[^ ^43^, ^44^ ^]^ our RuO_2_ anode formed by the coarse RuO_2_ particles was found to be stable and survived a long‐term operation under the low‐catalytic‐site utilization state for 120 h (**Figure** **5**a). This is reasonable, as due to the lower exposed surface area of the catalyst, the corrosion during electrolysis reaction can be suppressed.^[^ ^10^ ^]^ However, the RuO_2_/Ag composites anodes exhibited overwhelming stability for operating under a high catalytic site utilization at 1.00 A cm^−2^, compared with the RuO_2_ anode counterpart, which failed after 10 h of operation, as shown in Figure 5b and S9, Supporting Information. The PEMWE fabricated with the RuO_2_ anode suffered a significant performance decay after running at 1.00 A cm^−2^ for only 7 h; then the performance was able to maintain for a period of time on the next platform. The staged and gradual failure of the RuO_2_ anode rather than a sudden complete failure also confirms the potential failure mechanism for the RuO_2_ catalyst layer featured with insufficient electronic conductivity. The loss of catalyst is likely to bring catalyst layer defects during the long‐term operation, which results in a progressively degraded electronic conductance of the catalyst layer. While for the two‐layer RuO_2_/Ag anode, its stability was proved by operating under both the small current density of 0.20 A cm^−2^ (with almost no performance decay after 120 h) and the high current density of 1.00 A cm^−2^ (with a small potential increase by 13.3% after 100 h). The potential fluctuation during the long‐term operation at 1.00 A cm^−2^ mainly came from the temperature variation caused by the circulating water replenishment.

The improved durability of the PEMWEs fabricated with the RuO_2_/Ag composite anodes suggests that the Ag NWs compositing could benefit the electrode stability from two aspects. On the one hand, the presence of Ag in the anode may inhibit the overoxidation of RuO_2_ in OER due to the synergistic effect between RuO_2_ and Ag components.^[^ ^3^, ^45^, ^46^ ^]^ These kinds of metal/metal–oxide interactions have also been reported to prevent the Ir^3.2+^ catalytic sites from being oxidized to higher‐valent Ir^z+^ states by depositing on antimony‐doped tin oxide support, thus mitigating its dissolution during OER.^[^ ^47^ ^]^ To verify this, X‐ray photoelectron spectroscopy (XPS) measurements were performed to characterize the oxidation state changes of the RuO_2_ with and without the composition of Ag NWs after the durability test. Full‐scan X‐ray survey spectra verify the expected elements of Ru, O, S, F, and possible Ag in the catalyst layers (Figure S10–S12, Supporting Information). The 3d signal of Ag in the XPS spectrum is not quite obvious, as Ag is mostly buried under the RuO_2_ catalyst layer. As shown in Figure 5c, the characteristic Ru 3p spectra of the RuO_2_ anode involve doublet spin–orbit peaks at 462.4 and 484.3 eV, corresponding to the Ru 3p3/2 and Ru 3p1/2 orbit, respectively. Each 3p orbit of Ru can be further deconvoluted into two peaks, one is assigned to the Ru^4+^ of RuO_2_ and a satellite peak that can be assigned to the higher‐oxidized valence state of Ru^*n*+^.^[^ ^46^, ^48^ ^]^ For the RuO_2_ anode after the durability test, the Ru 3p peaks were observed to shift to a higher binding energy by 0.48 eV, indicating that the Ru valence increased during the long‐term OER process. An intensity decrease of the Ru 3p peaks was also observed after the long‐term operation, which was possibly due to the catalyst loss during water electrolysis. While with the incorporation of Ag in the composite anode, the valence states of Ru were basically maintained after the long‐term operation without obvious binding energy shift of the Ru 3p peaks, though the peak intensity decreased as well. The higher electronegativity of Ru (2.30) compared to that of Ag (1.93) determines it has stronger ability to attract electrons. During the electrochemical OER process, Ag could serve as a reducing agent to either inhibit the overoxidation and dissolution of RuO_2_, or the Ru oxidized to a higher valence state would be reduced by Ag on site. On the other hand, as water was circulated at the anode during the long‐term operation, the electrode performance degradation was also likely to come from the catalyst loss due to the erosion of water and the gas bubbles generation/release from the electrode.^[^ ^49^ ^]^ Figure 5d presents the retention rate of the RuO_2_ catalyst and its correlation with the performance loss of the PEMWEs fabricated with the RuO_2_/Ag composite anodes and the RuO_2_ anode after the long‐term durability test. We can find that the anodes participating in the water electrolysis in flowing aqueous media accompanied by the generation/release of a large amount of gas bubbles generally experienced severe catalyst loss after the long‐term operation, with the RuO_2_ retention rate of only 63%–66% at the end of the durability test. While for the RuO_2_/Ag composite anodes with the incorporation of Ag NWs to provide additional electronic conductance pathway, the catalytic sites utilization was ensured within the catalyst layer to enable the long‐term durability not only for the operation at small current density, but also at high current density. In the future research toward the development of anodes in PEMWEs, how to improve the firmness of the catalyst and optimize the electronic conductance within the catalyst layer is of great research significance to push forward the practical application of the PEMWE technology for green hydrogen production.

## 3 Conclusion

We have reported the fabrication of RuO_2_/Ag composite anodes based on the commercial submicron RuO_2_ catalyst, and the anodes with Ag NWs compositing exhibited optimized catalytic site utilization due to the improved electronic conductance within the catalyst layer, as well as improved mass transportation due to the highly porous feature of the Ag NW layer. The PEMWE fabricated with the two‐layer RuO_2_/Ag anode was able to deliver an electrolysis current of 1.77 A cm^−2^ at 2.00 V, which was increased by 34 % of that fabricated with the RuO_2_ anode, due to the enhanced electrode reaction kinetic and the improved mass transportation. The influences of both the morphology and the compositing way of Ag for forming the catalyst layer were investigated to demonstrate the role of Ag NWs in promoting the water electrolysis performance in PEMWEs. In particular, the long‐term stability of the RuO_2_/Ag composite anodes has been proved under the PEMWE operating conditions. Almost no performance decay of the PEMWE with the two‐layer RuO_2_/Ag anode was observed after running at 0.20 A cm^−2^ for 120 h, and the durability for operating under a high current density of 1.00 A cm^−2^ was greatly extended. The incorporation of Ag NWs was not only found to improve the catalytic site utilization within the catalyst layer to ensure the long‐term durability for operating under high current density, but also inhibit the peroxidation and dissolution of RuO_2_ during the acidic OER process due to the synergistic effect between Ag and RuO_2_. This work may provide some hints on the future development of highly effective and durable anodes in PEMWEs.

## 4 Experimental Section

4.1

4.1.1

##### CCM Fabrication

Nafion 117 membrane with the thickness of 183 μm and Chemours Nafion ionomer dispersion (D521, 1100 EW, 5 wt%) was bought from Fuel Cell Store, America. Ag NW dispersion (in ethanol, 20 mg mL^−1^, 60 nm in diameter, 10 μm in length) was bought from Nanochem, China. Ag powder (2–3.5 μm) was bought from Sigma‐Aldrich. RuO_2_ catalyst (Sigma‐Aldrich, 99.9% trace metals basis) was used as the anode catalyst, and platinum carbon (Pt/C, HiSPEC 8000, nominally 50 wt%, Alfa Aesar) was used as the cathode catalyst. All the CCMs were fabricated with the same Pt/C cathode. First, 20 mg Pt/C catalyst was mixed with ionomer and solvent solution in a glass vial to form a uniformly dispersed catalyst ink. The weight ratio of ionomer to catalyst (I/C ratio) was controlled to be 10 wt%. 2 mL solvent solution (with a formula of water to n‐propanol at 1:2, v/v) was then added. The vial containing the catalyst mixture was sealed with parafilm and then placed in ultrasonic water bath kept at a constant room temperature for 30 min prior to use. The well‐dispersed catalyst ink was immediately spray deposited onto a piece of membrane with a demarcated area of 2 × 2 cm^2^ to form a uniform catalyst layer using a manual spray gun (TAIWAN, HD‐131, with the caliber of 0.2 mm). Pt/C was used as the cathode catalyst at a constant loading of 1.2 mg cm^−2^ to minimize the influence of cathode HER polarization during the overall water electrolysis. The drying temperature during the spraying process was controlled to be 95 °C by a hot plate (RCT basic IKAMAG), and the spray passes and the movement rate were kept as stable as possible to reduce the errors caused by the spraying technique.

Different anodes were fabricated for investigation. First, the RuO_2_ anode was prepared as a control with the following procedures: 10 mg of RuO_2_ catalyst was mixed with the ionomer (at an I/C ratio of 10 wt%) and uniformly dispersed in 2 mL solvent (in equal proportion with that for cathode ink) to form the anode catalyst ink, which was then used to form the anode catalyst layer with the same spray coating processes. For the fabrication of the RuO_2_/Ag composite anodes, Ag NWs or Ag NPs (at a mass ratio of 1:4 to RuO_2_) were mixed with ionomer and introduced to the anode in different ways. Note that although the anode configuration was changed by introducing Ag NWs, the I/C ratio was kept constant to guarantee the same ionic conductivity within the catalyst layers. The catalyst loadings at both the anode and the cathode were determined by weighing the membrane weight gain before and after the deposition.

##### Electrode Characterizations

The cross‐section structures of the as‐prepared anodes were characterized by field‐emission scanning electron microscopy (FESEM, ZEISS ULTRA 55, Germany) under an accelerating voltage of 10 kV with SE2 detector. The detailed morphologies of RuO_2_ catalyst and Ag NWs were further examined by TEM (FEI Titan ChemiSTEM) under 200 kV. The specific surface area of RuO_2_ catalyst was determined by BET method on Micromeritics TriStar II at 77 K. Before N_2_ adsorption, the samples were degassed at 120 °C under vacuum state on the equipped VacPrep 061. In situ visualization of gas bubbles generation and release from the anode surface was performed on Nikon ECLIPSE ME600 equipped with a 5× magnification lens. The applied current was set to a constant 50 mA for water oxidation on different anodes to produce O_2_ during the observation. In‐plane electronic conductivity of the catalyst layers was measured by the VDP method, with the details described in the Supporting Information. Inductively coupled plasma–optical emission spectroscopy (ICP–OES) measurements were conducted on PerkinElmer Optima 8300 to determine the catalyst retention after the durability test, by dissolving the RuO_2_ scraped from the anodes in ascorbic acid and hydrochloride matrix to prepare the sample solutions. XPS was performed to study the surface chemical state changes of the catalyst before and after the durability test.

##### Cell Assembly and Electrochemical Measurements

The as‐prepared CCMs with the active area of 2 × 2 cm^2^ were fabricated into a customized electrolyzer for performance tests (Figure S13, Supporting Information). Two fiber felts (0.35 mm thickness, SCI Materials Hub, China) were used as the porous transport layers at both the cathode and the anode, and two polytetrafluoroethylene gaskets with the thickness of 0.40 mm were matched to enable the sealing of the electrolyzer. Electrochemical measurements of the PEMWEs were conducted on a single‐channel potentiostat (Zahner, ZENNIUM, Germany), equipped with an external high‐current power potentiostat (Zahner PP211, 10 A). Before the performance tests, ultrapure water was preheated to 80 °C and supplied to both the cathode and the anode to heat the electrolyzer up, and both the cell temperature and the circulating water temperature were kept at 80 °C during the performance tests. The electrolyzer was placed in a thermal‐insulating jacket to reduce the heat exchange with environment. Cyclic voltammetry (CV) for several cycles between 1.20 and 2.50 V, at a scan rate of 50 mV s^−1^, was conducted to activate the electrodes before recording the cell performance curves. The polarization curves were obtained by conducting linear sweep voltammetry (LSV) from 1.20 V to 2.20 V at a scan rate of 10 mV s^−1^. Steady‐state current density (*j*) responses under a set of potentials holding at each point for 5 min were recorded to obtain the Tafel plots. EIS measurements were performed under galvanostatic mode. An amplitude of 10% of the DC current was applied as the AC perturbation. The responses of impedance were recorded between 10 kHz and 100 mHz. Data points presented are averages of three measurements for per decade. The durability tests were conducted on IviumStat.h potentiostat (Ivium Technologies B.V., The Netherlands) by applying a constant current of 0.20 or 1.00 A cm^−2^ to the PEMWEs fabricated with the different anodes and recording the potential changes over time.

## Conflict of Interest

The authors declare no conflict of interest.

## Supporting information

Supplementary Material

## Data Availability

The data that support the findings of this study are available from the corresponding author upon reasonable request.
