# Supplementary material for: Silver Compositing Boosts Water Electrolysis Activity and Durability of RuO2 in a Proton‐Exchange‐Membrane Water Electrolyzer
Source: Small Sci. 2023 Jul 18;3(9):2300055. doi: 10.1002/smsc.202300055 (PMC11936012; doi:10.1002/smsc.202300055)
Supplement: Supplementary file 1 — Supplementary Material [file SMSC-3-2300055-s001.pdf]

Supporting Information

Silver Compositing Boosts Water Electrolysis Activity and Durability of RuO_2 in a Proton-Exchange-Membrane Water Electrolyzer

*Jiayi Tang, Yijun Zhong, Chao Su, and Zongping Shao**

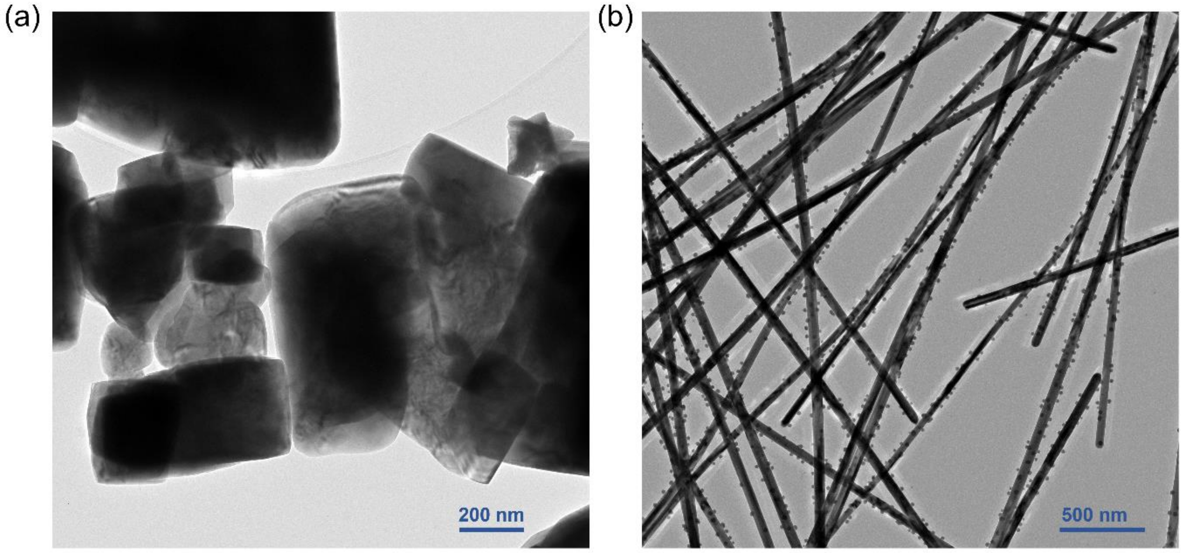

Figure S1. TEM images of the (a) commercial submicron RuO_2 nanoparticles, and (b) Ag NWs for fabricating the anodes in PEMWEs.

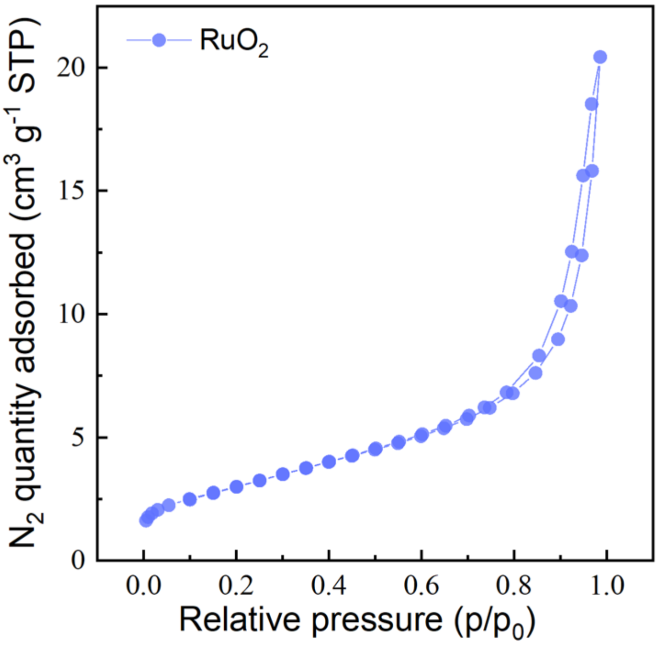

Figure S2. Nitrogen adsorption-desorption isotherm of the commercial submicron RuO_2 catalyst.

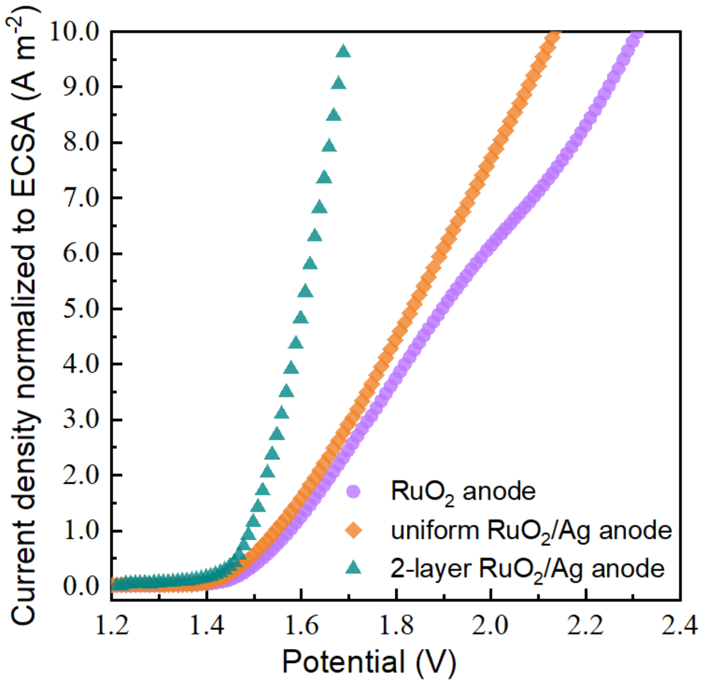

Figure S3. Polarization performance of the PEMWEs normalized to ECSA fabricated with RuO_2 anode, uniform RuO_2/Ag anode, and 2-layer RuO_2/Ag anode.

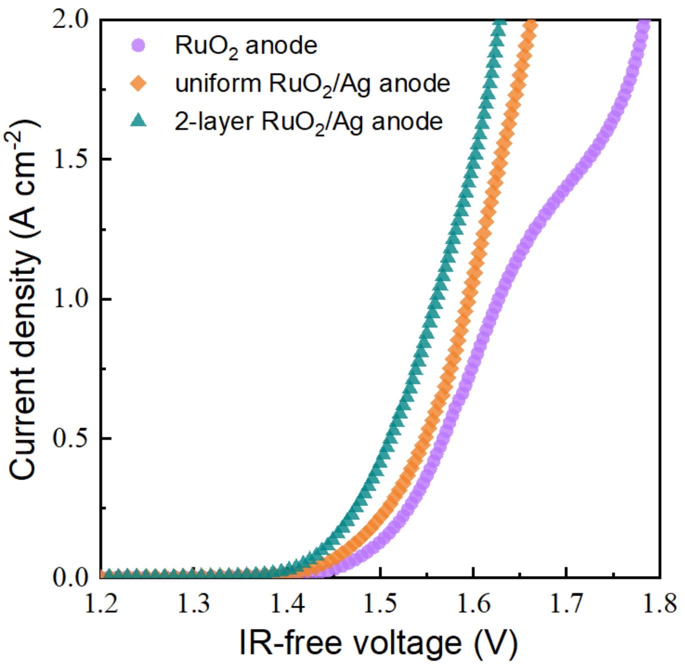

Figure S4. IR-free polarization performance of the PEMWEs fabricated with RuO_2 anode, uniform RuO_2/Ag anode, and 2-layer RuO_2/Ag anode.

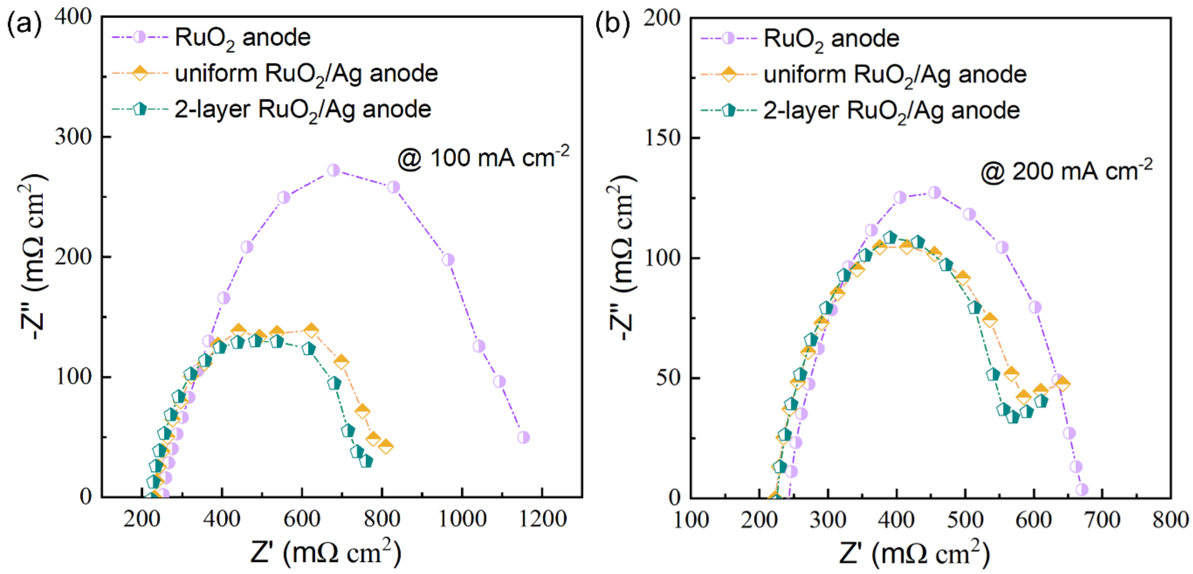

Figure S5. Nyquist plots of the PEMWEs operated at (a) 100 mA cm^{-2}, and (b) 200 mA cm^{-2}.

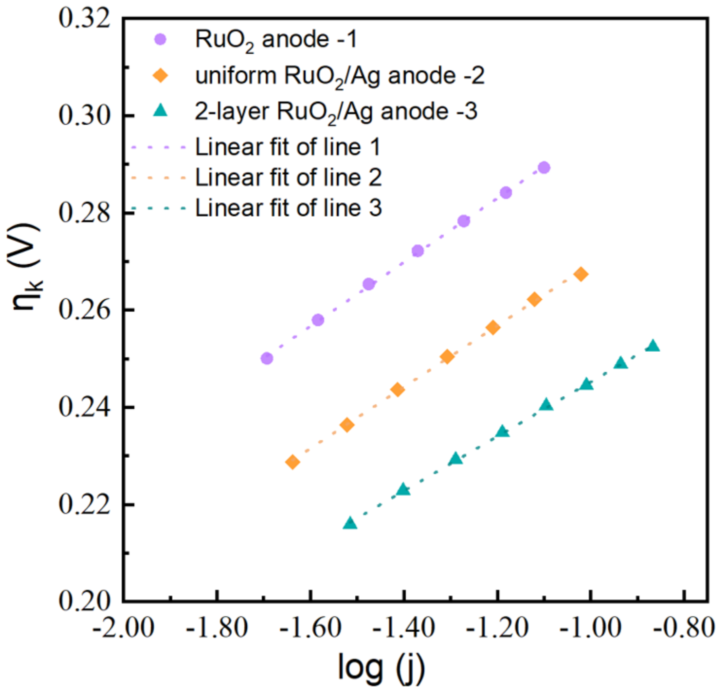

Figure S6. Tafel plots as-obtained based on the PEMWEs fabricated with different anodes. Each point was taken under steady state by recording the current density responses under a set of constant potentials. The kinetic fitting ranges for the RuO_2 anode, uniform RuO_2/Ag anode, and 2-layer RuO_2/Ag anode are 1.43-1.47 V, 1.41-1.45 V, and 1.39-1.43 V, respectively.

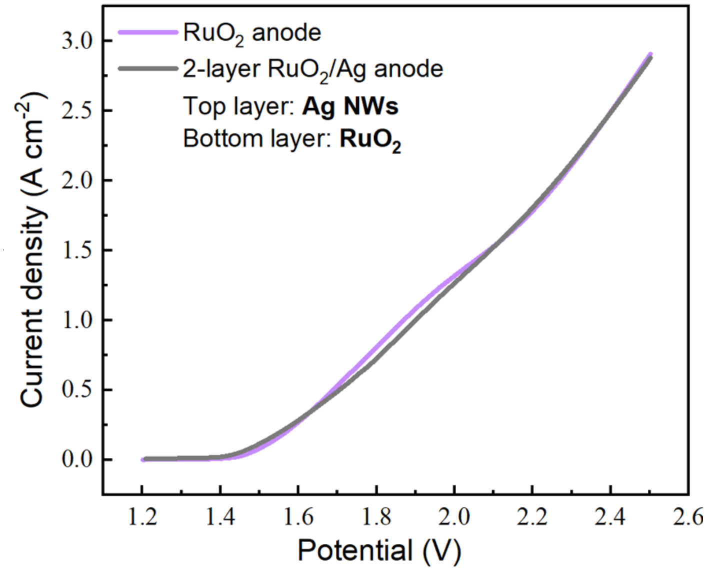

Figure S7. Polarization curves of the PEMWEs fabricated with RuO_2 anode, and the other 2-layer RuO_2/Ag composite anode with RuO_2 catalyst layer as the base layer and Ag NWs layer as the top layer.

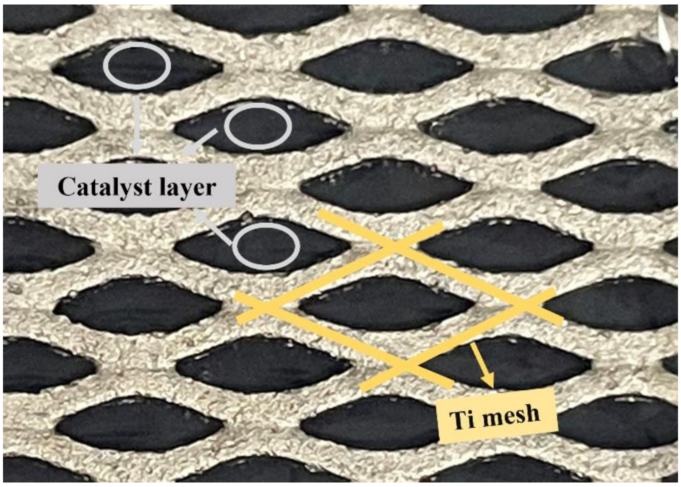

Figure S8. A front-view picture of the open anode setup for the in-situ observation of gas bubbles. A high-speed camera equipped with a 5× magnification lens is placed above to capture the generation and diffusion process of the O_2 bubbles during water electrolysis under the applied current. (Ti mesh with straight pores was pressed on the catalyst layer, and served as an applicator of the external electrical signal. The electrode was wholly soaked in water during the observation.)

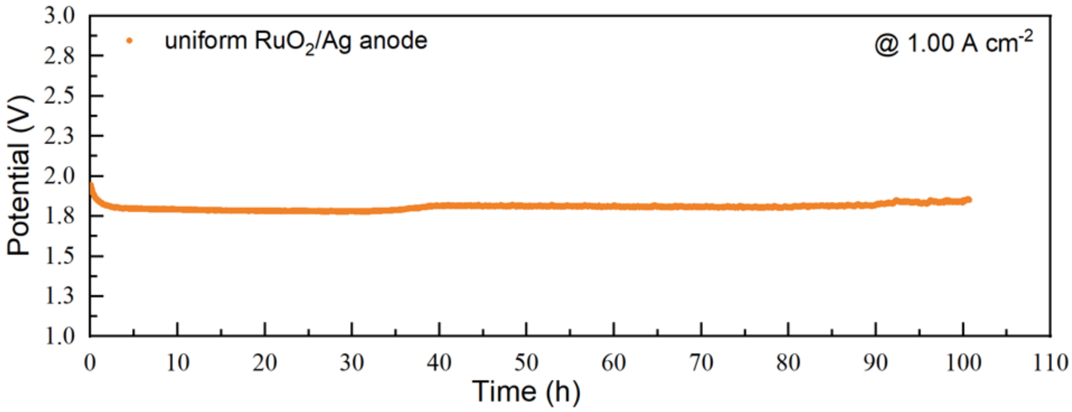

Figure S9. Durability test of the PEMWE fabricated with the uniform RuO_2/Ag anode by operating at 1.00 A cm^{-2} for 100 h.

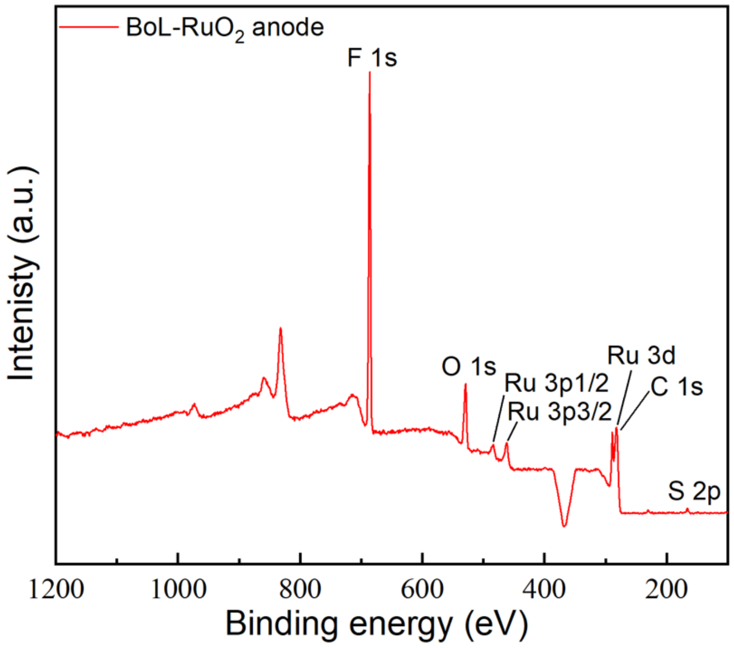

Figure S10. Full-scan X-ray survey spectrum of the RuO_2 anode at BoL of the 120-h durability test at 0.20 A cm^{-2}.

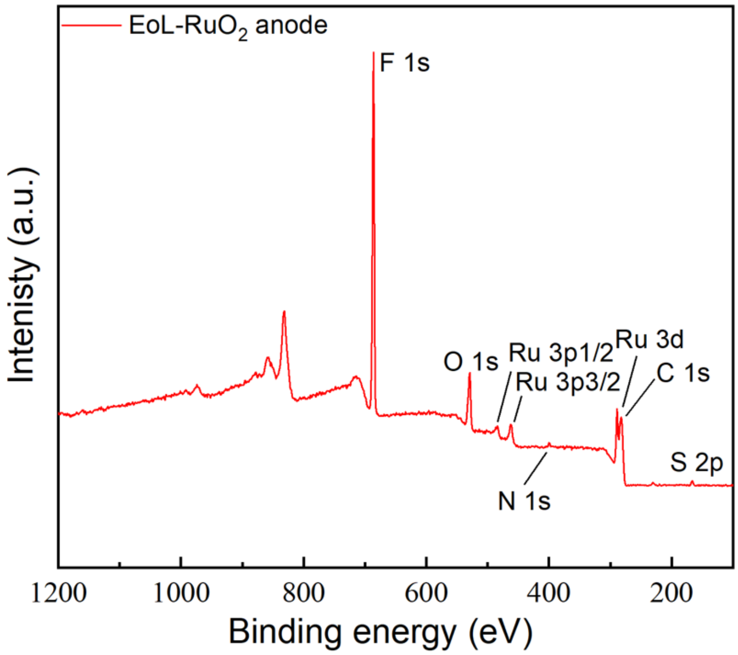

Figure S11. Full-scan X-ray survey spectrum of the RuO_2 anode at EoL of the 120-h durability test at 0.20 A cm^{-2}.

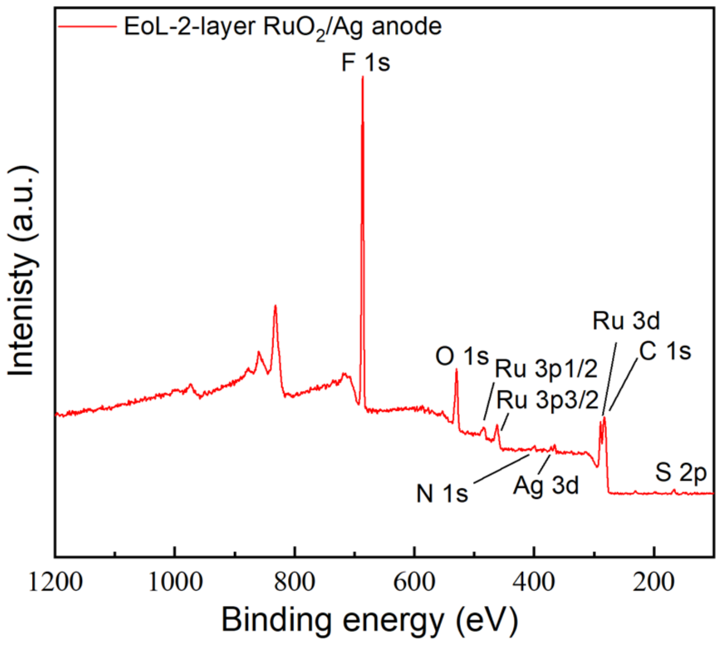

Figure S12. Full-scan X-ray survey spectrum of the 2-layer RuO_2/Ag anode at EoL of the 120-h durability test at 0.20 A cm^{-2}.

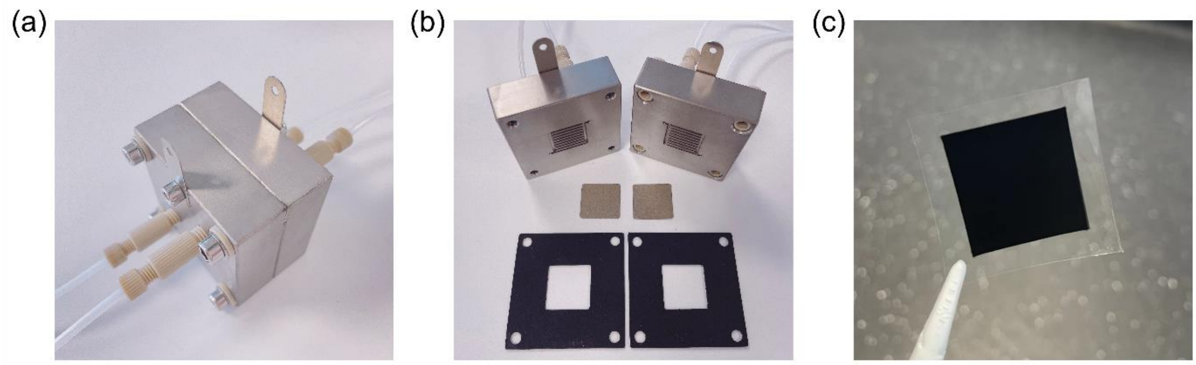

Figure S13. Photos of (a) the customized Ti-plate electrolyzer cell, and (b) the cell components as disassembled: a couple of Ti plates engraved with parallel flow fields, fluorine rubber sealing gaskets, and Ti fiber felts as the porous transport layer; (c) the as-prepared CCM by spraying coating method with uniform catalyst layers.

Table S1. A comparison of PEMWEs performance by applying Ir- or Ru-based anodes in regards of the precious metal loading.

Anode catalyst	Precious metal loading	PEMWE performance	Test conditions	Ref.
2-layer RuO_2/Ag anode (Commercial RuO_2)	0.375 mg_{Ru} cm^{-2}	1.08 A cm^{-2}@1.8 V<br>1.77 A cm^{-2}@2.0 V	N117, 80 °C, pure water	This work
RuO_2/Sb-doped SnO_2	1.5 mg_{Ru} cm^{-2}	1.0 A cm^{-2}@1.56 V	N212, 60 °C, deionized water	1
Ni-RuO_2	~ 2.2 mg $_{Ru}$ cm^{-2}	1.0 A cm^{-2}@1.95 V	N117, 0.1 M $HClO_4$, room temperature	2
Commercial IrO_2	1.0 mg_{Ir} cm^{-2}	1.0 A cm^{-2}@1.89 V	N117, 80 °C, pure water	3
$SnRuO_x$	1.46 mg_{Ru} cm^{-2}	1.0 A cm^{-2}@1.565 V	N212, 80 °C, distilled water	4
Ir/TiC	0.3 mg_{Ir} cm^{-2}	0.84 A cm^{-2}@1.80 V	N112, 80 °C, deionized water	5
90% IrO_2-ITO	2 mg_{Ir} cm^{-2}	1.0 A cm^{-2}@1.74 V	N115, 80 °C, deionized water	6
40Ir/TV-20	2.5 mg_{Ir} cm^{-2}	1.0 A cm^{-2}@2.0 V	N117, 80 °C, distilled water	7
40 % IrO_2/ antimony-doped tin oxide	2 mg_{Ir} cm^{-2}	1.0 A cm^{-2}@1.96 V	N115, 80 °C, deionized water	8

[a)] Pt/C catalysts are used as the cathode for all examples.

Table S2. Tafel slopes and exchange current densities as calculated from the Tafel plots.

Sample	Tafel slope [mV dec^{-1}]	Exchange current density [A cm^{-2}]
RuO_2 anode	65.8	3.1×10^{-6}
uniform RuO_2/Ag anode	63.1	5.4×10^{-6}
2-layer RuO_2/Ag anode	56.1	4.2×10^{-6}

Calculation of the overpotentials in the operating PEMWEs in Figure 2.

Under a certain electrolysis current, the cell voltage is mainly determined by the reversible cell potential (E_{rev}), as well as the overpotentials from ohmic loss (η_{Ohmic}), mass transport loss (η_{mass}), and the kinetic losses of the anode OER (η_{An}) and the cathode HER (η_{Ca}). Thus, the overall cell potential (E_{cell}) is expressed by the following equation:

$$E_{cell} = E_{rev} + \eta_{Ohmic} + \eta_{mass} + \eta_{An} + \eta_{Ca} \qquad (1)$$

Due to the sluggish OER kinetic, the overpotential of OER in acidic environment is typically

an order of magnitude higher than that of HER on Pt catalyst.[9] Besides, in this work, we used the same Pt/C cathode, thus, we ignore the effect of HER kinetic loss at the cathode in our analysis, and approximate the total kinetic loss (η_k) with the η_{An}, and simplify the equation to:

$$E_{cell} = E_{rev} + \eta_{Ohmic} + \eta_{mass} + \eta_k \qquad (2)$$

In presence of liquid water at the electrode, the activity of H_2O can be approximated as unity. The activities of gaseous H_2 and O_2 products are proportional to their partial pressures.[10] The reversable E_{rev} for water electrolysis is approximately in linear relationship with the operating temperature, and can be calculated from the equation below.[11] According to the Nernst equation, the thermoneutral potential (U_T) that required for enthalpy of the electrochemical decomposition of liquid water at 80 °C is 1.472 V.[12] At an operating temperature of 80 °C (353.15 K), the value of E_{rev} was calculated to be 1.183 V.[13, 14]

$$E_{rev} = \frac{1}{2F}\left(-159.6\frac{J}{K\,mol}T + 2.847 \times 10^5\frac{J}{mol}\right) \qquad (3)$$

The voltage loss from the ohmic resistance (η_{Ohmic}) of the cell at different current densities (i) can be calculated from the equation 4. The high frequency resistance (HFR) was obtained from EIS measurements.

$$\eta_{Ohmic} = i \times HFR \qquad (4)$$

The kinetic loss (η_k) is generally obtained from the Butler−Volmer equation 5, assuming that the polarization loss is mainly from the anode OER process. At the kinetic-controlled electrode reaction region, by plotting the iR drop corrected overpotential against the log (j), the kinetic governing parameters, Tafel slope (2.303RT/4F) and exchange current density (j^0) were obtained.

$$\eta_k = \left(\frac{RT}{\alpha nF}\right)\log(j^0) - \left(\frac{RT}{\alpha nF}\right)\log(j) \qquad (5)$$

The mass transport loss is a sum of water liquid, gases, and ions transfer behaviors at the electrode interface. It is hard to characterize individually, thus it is calculated by subtracting the losses from the other three parts.

Calculation of the in-plane electronic conductivity of the catalyst layers in Figure 3.

The in-plane electronic conductivity (σ_{IP}) of the catalyst layers is calculated as follows, by applying the VDP method, where t is the sample thickness, and R_s is defined as the sheet resistance of the catalyst layer in equation 6. First, the resistances were measured in two perpendicular directions, between point A, and B, as well as point A, and C, then the perpendicular resistances in AB and AC directions (denoted as R_{AB} and R_{AC}) were obtained and iterated into equation 7 for the calculation of the R_s.

$\sigma_{IP} = 1/R_s t$ (6)

$\exp\left(\frac{-\pi R_{AB}}{R_S}\right) + \exp\left(\frac{-\pi R_{AC}}{R_S}\right) = 1$ (7)

References

[1] X. Wu, K. Scott, *Int. J. Hydrog. Energy* **2011,** *36*, 5806-5810.

[2] Z.-Y. Wu, F.-Y. Chen, B. Li, S.-W. Yu, Y. Z. Finfrock, D. M. Meira, Q.-Q. Yan, P. Zhu, M.-X. Chen, T.-W. Song, Z. Yin, H.-W. Liang, S. Zhang, G. Wang, H. Wang, *Nat. Mater.* **2023,** *22*, 100-108.

[3] Y. N. Regmi, E. Tzanetopoulos, G. Zeng, X. Peng, D. I. Kushner, T. A. Kistler, L. A. King, N. Danilovic, *ACS Catal.* **2020,** *10*, 13125-13135.

[4] Z. Shi, J. Li, Y. Wang, S. Liu, J. Zhu, J. Yang, X. Wang, J. Ni, Z. Jiang, L. Zhang, Y. Wang, C. Liu, W. Xing, J. Ge, *Nat. Commun.* **2023,** *14*, 843.

[5] L. Ma, S. Sui, Y. Zhai, *Int. J. Hydrog. Energy* **2009**, *34*, 678-684.

[6] V. K. Puthiyapura, S. Pasupathi, H. Su, X. Liu, B. Pollet, K. Scott, *Int. J. Hydrog. Energy* **2014,** *39*, 1905-1913.

[7] C. Hao, H. Lv, C. Mi, Y. Song, J. Ma, *ACS Sustain. Chem. Eng.* **2016,** *4*, 746-756.

[8] V. K. Puthiyapura, M. Mamlouk, S. Pasupathi, B. G. Pollet, K. Scott, *J. Power Sources* **2014,** *269*, 451-460.

[9] D. Choudhury, R. Das, A. K. Tripathi, D. Priyadarshani, M. Neergat, *Langmuir* **2022,** *38* (14), 4341-4350.

[10] K. S. Schmitz, *Phys. Chem.*, Elsevier, Boston, **2017**.

[11] I. Barin, G. Platzki, *Thermochemical Data of Pure Substances*, Wiley, **1989**.

[12] C. Lamy, P. Millet, *J. Power Sources* **2020,** *447*, 227350.

[13] S. Chatterjee, X. Peng, S. Intikhab, G. Zeng, N. N. Kariuki, D. J. Myers, N. Danilovic, J. Snyder, *Adv. Energy Mater.* **2021,** *11* (34), 2101438.

[14] X. Peng, P. Satjaritanun, Z. Taie, L. Wiles, A. Keane, C. Capuano, I. V. Zenyuk, N. Danilovic, *Adv. Sci.* **2021,** *8* (21), 2102950.
